# Alleged Abortion…Inquest by the Coroner

**Published:** 1859-10

**Authors:** 


### *Alleged Abortion.—Inquest by the Coroner.*

Coroner Schirmer held an inquest upon the body of Mary E. Visscher, a native of Albany, thirty-three years of age, whose death was the result of an abortion, alleged to have been produced by Mrs. Elizabeth Byrnes, a female physician, fifty-five years of age. The evidence went to show that the deceased came to the house of Mrs. Byrnes, No. 168 Thompson street, on the 23d inst.

Dr. Geo. B. Bouton made a post-mortem examination of the body, and found that death had been caused by peritonitis, induced by a miscarriage and the violence used to produce the miscarriage. From the appearance of the uterus, he thought that the fœtus could not have been expelled more than forty-eight hours previous to the examination, which took place twelve hours after death.—(Mrs. Byrnes and nurse each testified that it was expelled on the 27th.) The case was then given to the jury, who rendered the following verdict: "That deceased came to her death by an abortion at the hands of Elizabeth Byrnes, and we consider Mary Smith an accessory before the act."

[*N. Y. Med. Express.*

